# Neutrophil myeloperoxidase index in juvenile dogs with parvoviral enteritis

**DOI:** 10.1177/10406387261436360

**Published:** 2026-04-04

**Authors:** Louis C. Loubser, Willem J. Botha, Yolandi Rautenbach, Anri Celliers

**Affiliations:** Department of Companion Animal Clinical Studies, University of Pretoria, Pretoria, South Africa; Panorama Veterinary Clinic and Specialist Centre, Cape Town, South Africa; Department of Companion Animal Clinical Studies, University of Pretoria, Pretoria, South Africa; Panorama Veterinary Clinic and Specialist Centre, Cape Town, South Africa; Department of Companion Animal Clinical Studies, University of Pretoria, Pretoria, South Africa; IDEXX Laboratories, Johannesburg, South Africa; Department of Companion Animal Clinical Studies, University of Pretoria, Pretoria, South Africa; Department of Clinical Sciences, College of Veterinary Medicine, Kansas State University, Manhattan, KS, USA

**Keywords:** canine parvovirus, cortisol, C-reactive protein, myeloperoxidase index, total thyroxine

## Abstract

Canine parvovirus is one of the most common causes of infectious enteritis in puppies worldwide. Although various biomarkers have been evaluated to predict prognosis in dogs with parvoviral enteritis (CPE), many are not feasible in routine practice. The neutrophil myeloperoxidase index (MPXI; derived from the Advia 2120 hematology analyzer, Siemens), has shown promise as a marker of inflammation and disease severity in other species. We compared the MPXI in 47 client-owned dogs with established prognostic indicators, including total WBC, neutrophil and lymphocyte count, and serum concentrations of total thyroxine, cortisol, and C-reactive protein (CRP). The MPXI in dogs with CPE did not differ significantly from healthy controls when measured at admission (*p* = 0.444), at 24 h (*p* = 0.332), or at 48 h (*p* = 0.279) after admission. At 24 h after admission, MPXI had a strong positive correlation with serum cortisol (*r* = 0.87; *p* < 0.001) and CRP (*r* = 0.71; *p* = 0.003) concentrations and a strong negative correlation with WBC (*r* = −0.82; *p* < 0.001), neutrophil (*r* = −0.77; *p* < 0.001), and lymphocyte (*r* = −0.90; *p* < 0.001) counts, as well as serum thyroxine concentration (*r* = −0.78; *p* < 0.001). MPXI did not distinguish between diseased and healthy animals. However, increased MPXI in dogs with parvoviral enteritis may indicate the presence of immunoparalysis and be associated with a worse prognosis. Larger prospective studies, including mortality data, are warranted to evaluate MPXI as an accessible and cost-effective prognostic biomarker in CPE.

Canine parvovirus (**CPV**; family *Parvoviridae*, taxon species *Protoparvovirus carnivoran1*) remains a globally important cause of viral enteritis in young dogs.^4,19^ In the absence of virus-specific therapy, treatment of canine parvoviral enteritis (**CPE**) has been limited to supportive care, with mortality rates >90% in untreated cases.^33^ Early intravenous administration of a canine parvovirus monoclonal antibody may prevent mortality and reduce disease duration; however, its efficacy has not been directly compared with standard treatment protocols.^29^ Intensive in-patient management achieves survival rates >80%,^10,18,54^ but the associated costs stemming from prolonged hospitalization and intensive nursing care are substantial.^25,53^ As a result, outpatient treatment protocols offering 75–80% survival have been developed to improve accessibility.^53,60^ Nevertheless, the prognosis remains guarded in some cases, and financial constraints continue to influence treatment decisions, with some owners electing euthanasia.^25,33^

Prognostic biomarkers can help guide treatment decisions by informing owners and veterinarians about the expected outcomes and the cost-benefit of therapy vs. euthanasia. Several hematologic, biochemical, acute phase, cytokine, and endocrine markers (**Table 1**) have been evaluated for predicting disease severity, hospitalization duration, and survival. Of particular relevance to our study, persistently elevated cortisol concentrations are associated with increased mortality and prolonged hospitalization.^15,54^ Recovery of thyroxine levels over time has been linked to improved survival.^42,54^ Elevated C-reactive protein (CRP) concentrations are associated with poor outcomes and the development of the systemic inflammatory response syndrome (**SIRS**).^28,34,48^ Higher total WBC, neutrophil, and lymphocyte counts are predictive of survival.^3,13,15,18,48^

**Table 1.** Summary of prognostic biomarkers investigated in dogs diagnosed with canine parvoviral enteritis.

Prognostic marker	Finding	Ref.
Hematology		
Hematocrit	Anemia—decreased odds of survival	^10^
**WBC count**	>4.5 × 10^9^/L 24 and 48 h after admission; 100% PPV for survival	^18^
	Progressive increase associated with survival	^3^
	>3.2 × 10^9^/L 72 h after admission; 89.5% PPV for survival	^15^
	<3.4 × 10^9^/L on admission; 100% PPV for SIRS	^48^
**Neutrophil count**	≥3.0 × 10^9^/L 24 h after admission; 100% PPV for survival	^18^
	Progressive increase associated with survival	^3^
	>1.6 × 10^9^/L 72 h after admission; 88.9% PPV for survival	^15^
	Neutropenia/circulating band neutrophils associated with decreased neutrophil function	^13^
**Lymphocyte count**	≥1.0 × 10^9^/L 24 h after admission; 100% PPV for survival	^18^
	Progressive increase associated with survival	^3^
Mean platelet volume	>13.2 fL—92.9% PPV for SIRS	^48^
Platelet:neutrophil ratio	>70.5 predicts survival, with 100% sensitivity and 52.8% specificity	^56^
Platelet:lymphocyte ratio	>484 predicts survival, with 73.3% sensitivity and 77.8% specificity	^56^
Biochemistry		
Albumin	Hypoalbuminemia on admission = prolonged hospitalization <22.9 g/L on admission = 100% PPV for SIRS	^24,48^
Cholesterol	Hypocholesterolemia associated with death	^57,62^
Magnesium	Hypermagnesemia = 2.50 lower odds of survival	^10^
Ionized calcium	Hypercalcemia after 24 h of treatment = 10.7 higher odds for non-survival	^38^
Glucose	Hypoglycemia = 1.85 lower odds for survival for every 1 mmol/L decrease in blood glucose concentration	^10^
Acute phase proteins		
**CRP**	>142 mg/L = 100% PPV for SIRS	^48^
	>97.3 mg/L 24 h after hospitalization differentiate survivors from non-survivors, with a sensitivity of 86.7%, specificity of 78.7%, PPV of 50%, and NPV of 96%	^34^
	>92.4 mg/L upon admission had a sensitivity of 91% and specificity of 61% for differentiating between survivors and non-survivors	^28^
Cytokines		
TNF	Elevated TNF associated with death	^45^
Endocrine		
**Thyroxine**	Concentration is significantly lower in non-survivors compared with survivors at admission Increasing concentration during the first 48 h associated with survival	^54^
	Increasing concentration over the first 5 d of hospitalization associated with survival	^42^
**Cortisol**	Persistent elevation = poor prognosis >224 nmol/L 48 h after admission had a sensitivity of 75%, specificity of 100%, and PPV of 100% for death	^54^
	<46.6 nmol/L 72 h after admission had a sensitivity of 71.4%, specificity of 94.4%, and PPV of 89.5% for survival	^15^

NPV = negative predictive value; PPV = positive predictive value; SIRS = systemic inflammatory response syndrome; TNF = tumor necrosis factor. Markers investigated in our study are in bold.

Myeloperoxidase (**MPO**), a neutrophil primary granule enzyme, functions as a catalyst to produce hypochlorous acid, a powerful oxidizing agent, and forms an important part of the oxygen-dependent antimicrobial system of phagocytes.^26,59,63^ The Advia 2120 (Siemens) is a validated automated hematology analyzer that operates using 2 channels: the basophil and peroxidase channels.^17,37,43^ The basophil channel uses light scatter (cell size) and nuclear density measurements to generate the total WBC count.^17^ Following MPO staining, the peroxidase channel analyzes light scatter and light absorption to produce the differential WBC count and a scattergram of leukocyte clusters, with MPO staining on the x-axis and cell size on the y-axis.^17^ Using MPO staining data, the analyzer also calculates the myeloperoxidase index (**MPXI**). MPXI reflects the average neutrophil MPO content and is determined by measuring enzyme synthesis during neutrophil development versus depletion through activation and degranulation.^22,35,63^ MPXI is seen as a dynamic, context-dependent marker that is influenced by neutrophil maturation, activation, and degranulation; both increased and decreased MPXI values may reflect different phases or severities of systemic inflammation.

In human medicine, MPXI has been reported as a marker of neutrophil activation and systemic inflammation.^6,30,35^ One study found higher MPXI in septic patients compared with non-infectious SIRS.^9^ Another study found lower MPXI in bacterial sepsis than in non-septic bacterial conditions.^63^ The MPXI has also been used to distinguish between acute lymphoblastic and myeloblastic leukemia, as an aid in the diagnosis of megaloblastosis, and as a marker for the diagnosis and monitoring of ischemic heart disease complicated with arteriosclerosis obliterans.^20,21,64^

In contrast, little research has been done on MPXI as a marker of inflammation in domestic animals. In horses, MPXI did not differ between healthy, locally inflamed, and systemically inflamed individuals in one study; another study reported lower MPXI in horses with SIRS or sepsis, with the former being lower than the latter.^22,55^ Conversely, septic, neutropenic foals had increased MPXI, possibly attributed to impaired neutrophil activation and degranulation.^49^ In dogs, MPXI decreased 21 d after experimental *Ehrlichia canis* infection, which was attributed to persistent neutropenia or lower neutrophil MPO content resulting from degranulation or maturation abnormalities.^16^ Dogs naturally infected with *Babesia rossi* had significantly increased MPXI, especially non-survivors, possibly indicating immunoparalysis in cases with a more severe inflammatory response.^8^ Acquired MPO deficiency has been associated with diseases that cause severe leukocyte consumption. Dogs with CPE had a significantly lower MPXI compared with dogs with other gastrointestinal or inflammatory diseases.^27^ Increased MPXI has been described in non-surviving CPE cases during hospitalization, particularly on day 4, although these findings remain unpublished.^58^

To our knowledge, MPXI has not been specifically characterized and compared with other prognostic biomarkers in dogs with CPE. A literature search of PubMed and Google Scholar using the terms “canine parvovirus”, “myeloperoxidase index”, “MPXI”, “neutrophil myeloperoxidase”, and “dog” yielded no studies describing this relationship. Our objectives were to compare MPXI between CPV-infected dogs and healthy controls and, within the CPV-infected group, correlate MPXI with survival status and with previously identified prognostic biomarkers, including WBC, neutrophil, and lymphocyte counts, and serum total thyroxine, cortisol, and CRP concentrations. We hypothesized that MPXI would be significantly lower in CPV-infected dogs compared with controls. Additionally, within the CPV-infected group, we hypothesized that higher MPXI values would be correlated positively with survival and with the prognostic indicators (namely WBC, neutrophil, and lymphocyte counts, and serum total thyroxine concentration) and negatively with serum cortisol and CRP concentrations.

## Materials and methods

We planned a multi-center, prospective, cohort study of dogs naturally infected with CPV that were presented to the Panorama Veterinary Clinic and Specialist Centre (PVC; Cape Town, South Africa) and the Onderstepoort Veterinary Academic Hospital, University of Pretoria (OVAH; Pretoria, South Africa), from 2023 February to 2024 October. However, because of low prospective case recruitment, we retrospectively analyzed the OVAH medical records of dogs that were diagnosed with CPE that had data and samples collected and stored as part of previous CPE projects between 2018 and 2019. Control cases were included retrospectively to follow the reduction principle for conducting ethical research. Our study (REC 115–22) and the previous CPE projects (V048-18, V073-18, V090-18) received ethical approval from the University of Pretoria Animal Ethics Committee. Written owner consent was acquired prior to enrollment of all dogs.

For the prospective component of our study, the sample size was estimated based on a study of MPXI in 8 dogs with CPE,^27^ and an estimated survival probability of 70%. The sample size was estimated based on comparing mean MPXI values in surviving versus non-surviving puppies. Non-surviving puppies were assumed to have a x̄ ± SD MPXI of −0.6 ± 7.2, based on data from all 8 dogs. Surviving puppies were assumed to have a x̄ ± SD MPXI of 5.0 ± 4.2, based on data from 4 dogs with higher-than-median MPXI values in the previous publication.^27^ Calculations yielded a total requirement of 23 surviving and 17 non-surviving puppies. The comparison of expected mean MPXI in healthy puppies^37^ to those with CPE required a sample size of at least 3 dogs. However, we increased this number to 10 because control cases were not prospectively enrolled or matched to the population characteristics of the infected group.

**Figure 1.**
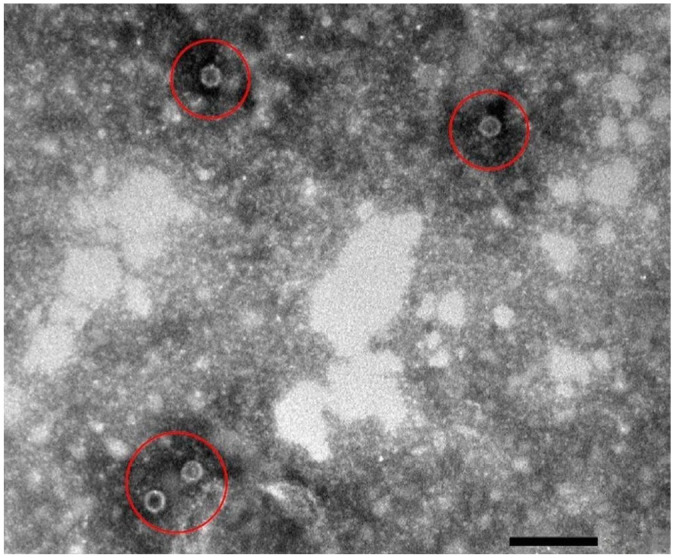
Electron micrograph of a fecal sample of a prospectively enrolled case with canine parvovirus particles (circles). Bar = 100 nm.

Inclusion criteria for both the prospectively and retrospectively recruited CPE dogs included any breed or sex between 6-wk- and 12-mo-old with a weight of at least 3 kg. Dogs had to have at least one clinical sign of CPV infection, which included depression, vomiting, diarrhea, anorexia, and/or dehydration. Peripheral blood smears had to be negative for blood-borne parasites. A diagnosis of CPE was made using a commercial antigen ELISA (SNAP parvo test; Idexx). Infection was confirmed via fecal electron microscopy (EM; CM10 transmission electron microscope, Philips; **Fig. 1**), which was also performed to concurrently screen for other enteric viral pathogens. Additionally, because of the severity of their clinical signs, prospectively recruited dogs were admitted to the PVC or OVAH isolation wards for treatment. Retrospectively recruited dogs had to have a CBC performed on the Advia 2120 at the time of diagnosis and sufficient serum stored at −80°C in the Onderstepoort Veterinary Clinical Pathology Laboratory and Biobank (OVCPLB; Pretoria, South Africa) to allow for CRP, total thyroxine, and cortisol measurement. For both groups, any treatment with antibiotic, corticosteroid, or other anti-inflammatory medication before admission led to exclusion from the study.

The controls were 10 client-owned dogs that were presented to the OVAH for routine vaccination or sterilization. Dogs had to be between 6-wk- and 12-mo-old, of any breed or sex, and weigh at least 3 kg. They had to have a CBC performed on the Advia 2120 at the time of evaluation and sufficient serum stored in the OVCPLB to allow for CRP, total thyroxine, and cortisol measurement. Dogs were deemed healthy based on history, full clinical examination, peripheral blood smear evaluation, CBC, and fecal EM to exclude CPV and other viral pathogens. Criteria for exclusion included a history of illness or antibiotic, corticosteroid, or other anti-inflammatory medication treatment within the preceding 2 wk. Healthy dogs kept on the same premises as other CPE-infected dogs were also excluded.

For the prospective cohort, a fecal sample was collected upon admission and used to perform a fecal flotation (Kyron Laboratories), fecal wet preparation, and CPV ELISA (Snap test; Idexx). A portion of the sample was submitted for EM to confirm the presence of CPV and screen for other enteric viral pathogens. Blood was collected via jugular venipuncture into EDTA and serum tubes (BD Biosciences) upon admission (T0; prior to initiation of treatment), 24 h (T1), and 48 h (T2) after initiation of treatment, because previous studies have shown that leukocyte parameters and serum cortisol, thyroxine, and CRP concentrations measured within this period may aid in predicting prognosis.^15,18,28,34,54^ The EDTA sample was used for a CBC (Advia 2120). The EDTA samples collected from dogs at PVC were submitted to PathCare Vetlab (Pathcare Park, Cape Town, South Africa) to determine the CBC; samples collected at the OVAH were evaluated at the OVCPLB. At both sites, these samples were submitted and analyzed within 12 h.

Before the study, Streck external quality control (**EQC**) data of the 2 Advia 2120 analyzers (PathCare and OVCPLB) were used to calculate the observed total error (TEobs), sigma metrics, and quality goal index for each quality control material level (low, medium, high). Thereafter, comparability of test results was assessed between the 2 analyzers using the American Society of Veterinary Clinical Pathology (ASVCP) total allowable error (TEa) for hematology measurands.^1,7,41^ Serum samples were used to determine thyroxine, CRP, and cortisol concentrations. After allowing blood samples to clot, and within one hour of collection, the tubes were centrifuged and serum was harvested. Serum samples collected at PVC were stored at 4°C for no longer than 5 d (during which time the samples were couriered to the OVCPLB), then stored at −80°C until batch analysis. Serum samples collected at the OVAH were immediately stored at −80°C until batch analysis. Serum thyroxine and cortisol concentrations were determined (Immulite 2000 analyzer; Siemens). CRP was determined (Cobas Intergra 400 Plus analyzer; Roche). Dogs admitted to the PVC or OVAH isolation wards received in-patient treatment according to a standard protocol, which included intravenous crystalloid or colloid fluid therapy, antibiotics, antiemetics, analgesic therapy with opioids, and glucose and potassium supplementation as needed, as well as enteral nutrition. The duration of hospitalization and outcome of treatment (discharge, death, or euthanasia) were recorded for each patient.

Data collected for the controls and retrospective cohort included signalment, WBC, neutrophil, and lymphocyte count, MPXI, and CRP concentration at the times of admission and diagnosis. Total thyroxine and cortisol concentrations were retrospectively measured on stored serum samples. The retrospective cases were either treated on an outpatient basis (because owners declined in-patient treatment, resulting in inconsistent follow-up times and some cases being lost to follow-up) or were hospitalized for treatment (data and/or samples were not collected on subsequent days of hospitalization and stored for evaluation, as this was not required for the previous CPE projects). Therefore, only data and/or samples collected at the time of diagnosis (T0; before any treatment was administered) were considered for further analysis.

For data analysis, the study population was divided into control, retrospective CPV-infected cases, and prospective CPV-infected cases. For analysis of data at T0, prospective and retrospective CPV-infected cases were grouped. For comparison of data among T0, T1, and T2 and for the estimation of correlations, only data from the prospective cohort was considered. The prospective cases were further divided into survivors and non-survivors (death or euthanasia). In the event of euthanasia, the reason for euthanasia was recorded. Statistical analysis was performed (SAS v.9.4) and evaluated at the 5% level of significance. Laboratory data were assessed for normality by evaluating descriptive statistics, plotting histograms, and performing normality tests. Laboratory data were compared between CPV-infected dogs and controls using non-parametric analysis, including the Mann–Whitney U test and Fisher exact test in the event of numerical and categorical data, respectively. Data from prospectively recruited CPV-infected dogs were compared at various times using Wilcoxon signed-rank test. Correlations between laboratory data were estimated by Spearman rank correlations. To categorize the strength of associations, absolute values of *r* were interpreted as very weak (0–0.19), weak (0.2–0.39), moderate (0.40–0.59), strong (0.6–0.79), and very strong (0.8–1.0). For analysis, dogs with a total thyroxine value of <6.4 nmol/L were assigned a value of 6.4 nmol/L, dogs with a cortisol value <27.6 nmol/L were assigned 27.6 nmol/L, and dogs with a CRP value of <10.0 mg/L were assigned 10.00 mg/L.

## Results

Hematology measurand results obtained from the 2 Advia hematology analyzers were comparable, when assessed against ASVCP hematology TEa recommendations.^7,41^

Between Feb 2023 and Oct 2024, 60 dogs with CPE were assessed for prospective enrollment. Forty-four dogs were excluded because of owner-declined hospitalization (*n* = 18), age (*n* = 11), low body weight (*n* = 9), or prior antibiotic use (*n* = 6). Of the 35 retrospective and 16 control cases assessed for eligibility, 4 and 6 cases, respectively, were excluded because of insufficient serum volume. The final study population included 16 prospective (PVC = 11; OVAH = 5) and 31 retrospective CPV-infected dogs, with 10 healthy controls. Differences were not significant between groups in age (*p* = 0.566), sex (*p* = 0.371), or weight (*p* = 0.470; **Table 2**).

**Table 2.** Demographic characteristics (sex, breed, age, and body weight) of dogs enrolled in our study.

Variable	Population			
	Controls (*n* = 10)	Prospective (*n* = 16)	Retrospective (*n* = 31)	Affected (prospective + retrospective) (*n* = 47)
Sex				
Female intact	2	5	8	13
Female spayed	1			
Male intact	6	11	21	32
Male castrated			1	1
Unknown	1		1	1
Breed				
American Pitbull Terrier		1	6	7
Beagle		1		1
Boerboel	3		3	3
Boxer			2	2
Bull Terrier	1			
Mixed breed	1	3	12	15
Dachshund	1	1		1
Dutch Shepherd		1		1
German Shepherd		1		1
Golden Retriever	1			
Great Dane		1		1
Jack Russell Terrier		1	2	3
Labradoodle		1		1
Labrador Retriever			2	2
Maltese		2		2
Rhodesian Ridgeback			1	1
Rottweiler		1	1	2
Scottish Terrier	1			
Siberian Husky	2	1		1
Standard Schnauzer		1		1
Unknown			1	1
Yorkshire Terrier	0		1	1
Age, mo (median; IQR)	3.5; 2.0–12.0	4.6; 1.5–11.0	4.0; 2.0–12.0	4.0; 1.5–12.0
Body weight, kg (median; IQR)	9.6; 3.0–25.6	9.5; 3.3–21.8	7.3; 3.6–21.7	8.1; 3.3–21.8

IQR = interquartile range.

Of the 16 prospective cases, 14 survived, and 2 died within 48 h of admission; none were euthanized. Prospective cases had a median duration of illness prior to presentation of 1 d (IQR; 1–2 d); this information was not recorded for retrospective cases. One prospective case lacked CBC data at T0 because of a technical laboratory error, but serum CRP, total thyroxine, and cortisol were measured. Two dogs died before T2, precluding data collection at that time. No significant differences in MPXI, WBC, neutrophil count, lymphocyte count, CRP, total thyroxine, or cortisol concentrations were found between prospective and retrospective CPV-infected dogs at T0 (**Table 3**).

**Table 3.** Comparison of MPXI and previously identified prognostic indicators between dogs affected by canine parvoviral enteritis and healthy controls at T0, T1, and T2.

Measurand	Controls, median (IQR)	Affected cases, median (IQR)							
		T0				T1		T2	
		Prospective	Retrospective	Combined	*p-*value	Affected	*p-*value	Affected	*p-*value
WBC count,* ×* 10^9^/L	11.6 (10.2–12.9)	8.5 (2.9–11.4)	6.2 (3.5–10.2)	7.3 (3.3–10.4)*	0.005	3.2 (1.1–8.9)*	<0.001	4.3 (2.8–8.1)*	<0.001
Neutrophil count,* ×* 10^9^/L	7.0 (6.4–8.0)	6.1 (1.8–9.8)	4.1 (0.9–8.7)	5.3 (1.6–9.2)	0.240	1.5 (0.3–6.4)*	0.027	1.6 (0.4–5.7)*	0.003
Lymphocyte count,* ×* 10^9^/L	3.4 (2.6–4.1)	0.8 (0.5–1.2)	0.7 (0.4–1.3)	0.7 (0.4–1.3)*	<0.001	1.02 (0.7–1.3)*	<0.001	1.6 (1.0–2.2)*	0.001
MPXI	19.9 (15.7–26.6)	18.0 (14.1–21.9)	18.5 (12.5–23.1)	18.2 (13.4–22.2)	0.444	25.2 (14.7–38.5)	0.332	15.6 (9.4–30.9)	0.279
CRP, mg/L	10.0 (10.0–10.0)	138 (112–175)	172 (91.5–205)	144 (94.3–195)*	<0.001	137 (118–185)*	<0.001	128 (71.5–180)*	<0.001
Total thyroxine, nmol/L	42.6 (33.3–47.5)	22.5 (9.8–29.6)	14.9 (6.4–21.8)	16.0 (8.3–23.8)*	<0.001	9.3 (6.4–30.4)*	0.001	12.0 (6.4–18.7)*	<0.001
Cortisol, nmol/L	56.8 (29.0–88.6)	185 (108–468)	118 (79.5–417)	145 (84.1–447)*	<0.001	169 (88.3–322)*	0.002	126 (51.0–258)*	0.002

CRP = C-reactive protein; IQR = interquartile range; MPXI = myeloperoxidase index; T0 = admission; T1 = 24 h; T2 = 48 h.

* = significant difference compared with controls.

**Figure 2.**
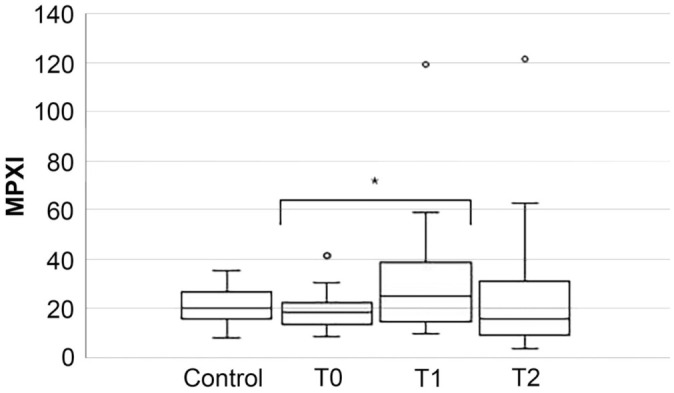
Box-and-whisker plot comparing myeloperoxidase index (MPXI) between control and prospectively affected dogs at presentation (T0), 24 (T1), and 48 (T2) h after hospitalization. In each plot, the box is the IQR, and the horizontal bar inside the box is the median. The T-bars denote the range; circles mark outliers. * = significant difference.

The median MPXI did not differ significantly between CPV-infected and control dogs at T0 (*p* = 0.444), T1 (*p* = 0.332), or T2 (*p* = 0.279; **Fig. 2**, Table 3). However, within the prospective cohort, MPXI increased significantly from T0 to T1 (*p* < 0.001), with no difference found between T0 and T2 (*p* = 0.954; **Table 4**).

**Table 4.** Comparison of MPXI and previously identified prognostic indicators between dogs in the prospective affected group at T0, T1, and T2 after hospitalization.

Measurand	T0, median (IQR)	T1		T2	
		Median (IQR)	*p-*value	Median (IQR)	*p-*value
WBC count, × 10^9^/L	8.5 (2.9–11.4)	3.2 (1.1–8.9)*	0.002	4.3 (2.8–8.1)*	0.020
Neutrophil count, × 10^9^/L	6.1 (1.8–9.8)	1.5 (0.3–6.4)*	0.001	1.6 (0.4–5.7)*	0.003
Lymphocyte count, × 10^9^/L	0.8 (0.5–1.2)	1.0 (0.7–1.3)	0.720	1.6 (1.0–2.2)*	0.004
MPXI	18.0 (14.1–21.9)	25.2 (14.7–38.5)*	<0.001	15.6 (9.4–30.9)	0.954
CRP, mg/L	138 (112–175)	137 (118–185)	0.821	128 (71.5–180)	0.808
Total T4, nmol/L	22.5 (9.8–29.6)	9.3 (6.4–30.4)	0.277	12.0 (6.4–18.7)*	0.022
Cortisol, nmol/L	185 (108–468)	169 (88.3–323)	0.175	126 (51.0–258)	0.173

CRP = C-reactive protein; IQR = interquartile range; MPXI = myeloperoxidase index; T0 = admission; T1 = 24 h; T2 = 48 h.

* = significantly different compared with T0.

CPV-infected dogs had significantly lower WBC counts at all times compared with controls (T0: *p* = 0.005; T1 and T2: *p* < 0.001; Table 3). Within the prospective cohort, the WBC decreased from T0 to T1 (*p* = 0.002) and T2 (*p* = 0.020; Table 4). The MPXI was correlated negatively with the WBC. The strongest correlations were seen between MPXI at T0 and WBC at T1 (*r* = −0.77; *p* = 0.001) and between MPXI and WBC, both at T1 (*r* = −0.82, *p* < 0.001; **Table 5**).

**Table 5.** The strength of correlation between MPXI and the prognostic indicators within the first 48 h after admission.

	MPXI					
	T0		T1		T2	
Measurand	*r-*value	*p*-value	*r*-value	*p*-value	*r*-value	*p*-value
WBC count						
T0	−0.37	0.010	—	—	—	—
T1	−0.77	0.001	−0.82	<0.001	—	—
T2	−0.42	0.131	−0.55	0.050	−0.55	0.043
Neutrophil count						
T0	−0.38	0.008	—	—	—	—
T1	−0.69	0.004	−0.77	<0.001	—	—
T2	−0.49	0.075	−0.59	0.034	−0.63	0.016
Lymphocyte count						
T0	−0.14	0.362	—	—	—	—
T1	−0.71	0.003	−0.90	<0.001	—	—
T2	−0.35	0.227	−0.50	0.082	−0.27	0.358
CRP						
T0	0.20	0.181	—	—	—	—
T1	0.59	0.018	0.71	0.003	—	—
T2	0.27	0.350	0.38	0.201	0.27	0.358
Total thyroxine						
T0	−0.25	0.088	—	—	—	—
T1	−0.65	0.007	−0.78	<0.001	—	—
T2	−0.51	0.062	−0.71	0.007	−0.20	0.503
Cortisol						
T0	0.27	0.070	—	—	—	—
T1	0.63	0.009	0.87	<0.001	—	—
T2	0.42	0.135	0.63	0.022	0.38	0.180

CRP = C-reactive protein; IQR = interquartile range; MPXI = myeloperoxidase index; T0 = admission; T1 = 24 h; T2 = 48 h; dash (—) = not calculated.

Neutrophil counts did not differ significantly between groups at T0 (*p* = 0.240) but were significantly lower in CPV-infected dogs at T1 (*p* = 0.027) and T2 (*p* = 0.003) compared with controls (Table 3). Within the prospective cohort, neutrophil counts significantly decreased from T0 to T1 (*p* = 0.001) and T2 (*p* = 0.003; Table 4). The MPXI had negative correlations with the neutrophil count. The strongest correlations were seen between the MPXI at T0 and neutrophil count at T1 (*r* = −0.69; *p* = 0.004) and between the MPXI and neutrophil count, both at T1 (*r* = 0.77, *p* < 0.001; Table 5). In addition, the MPXI and neutrophil count at T2 had a strong negative correlation (*r* = −0.63, *p* = 0.016; Table 5).

Lymphocyte counts were significantly lower in CPV-infected dogs at T0, T1 (both *p* < 0.001), and T2 (*p* = 0.001) compared with controls (Table 3). Within the prospective cohort, counts did not significantly differ between T0 and T1 (*p* = 0.720) but were significantly increased from T0 to T2 (*p* = 0.004; Table 4). The MPXI had strong negative correlations with lymphocyte count, especially between MPXI at T0 and lymphocytes at T1 (*r* = −0.71; *p* = 0.003), and between MPXI and lymphocytes at T1 (*r* = −0.90, *p* < 0.001; Table 5).

Serum CRP was significantly higher in CPV-infected dogs at T0, T1, and T2 (all *p* < 0.001) compared with controls (Table 3). No significant within-group differences were observed over time (Table 4). The MPXI was correlated positively with CRP, with the strongest correlations between MPXI at T0 and CRP at T1 (*r* = 0.59; *p* = 0.018), and MPXI and CRP, both at T1 (*r* = 0.71, *p* = 0.003; Table 5).

Thyroxine concentrations were significantly lower in CPV-infected dogs at T0 (*p* < 0.001), T1 (*p* = 0.001), and T2 (*p* < 0.001) compared with controls (Table 3). Within the prospective group, T0 and T1 values did not differ (*p* = 0.277), but thyroxine decreased significantly from T0 to T2 (*p* = 0.022; Table 4). The MPXI was correlated negatively with thyroxine, with the strongest correlations between MPXI and thyroxine, both at T1 (*r* = −0.78; *p* < 0.001), and MPXI at T1 and thyroxine at T2 (*r* = −0.71, *p* = 0.007; Table 5).

Cortisol concentrations were significantly higher in CPV-infected dogs at T0 (*p* < 0.001), T1, and T2 (both *p* = 0.002) compared with controls (Table 3). No significant within-group changes were observed over time (Table 4). The MPXI was correlated positively with cortisol, with the strongest correlation found at T1 (*r* = 0.87, *p* < 0.001; Table 5).

## Discussion

To our knowledge, the MPXI in dogs diagnosed with CPE has not been reported previously in comparison with previously identified prognostic biomarkers. We found that the MPXI did not significantly differ between affected and control dogs at diagnosis. However, MPXI increased significantly after 24 h of treatment. At that point, MPXI had a strong positive correlation with serum cortisol and CRP concentrations and a strong negative correlation with the WBC, neutrophil, and lymphocyte counts, as well as serum total thyroxine concentration.

In our study, the median MPXI measured upon admission did not differ significantly from that of our healthy control population. Contrary to our primary hypothesis, the MPXI increased significantly after 24 h of treatment. Significantly lower MPXI has been reported in dogs with CPE compared with those with other gastrointestinal or inflammatory diseases.^27^ Given that severe gastrointestinal inflammation results in marked neutrophil recruitment and activation,^12,51^ a decrease in MPXI would be expected, because MPXI reflects the balance between synthesis during promyelocyte maturation and neutrophil activation and degranulation.^63^ However, neither timing of MPXI measurement nor treatment protocols were described in the previous study,^27^ limiting direct comparison.

Our abovementioned MPXI findings have 5 possible explanations. First, margination of activated neutrophils with depleted MPO content during endotoxemia leaves relatively MPO-rich neutrophils in circulation, resulting in normal or increased MPXI values.^49^

Second, unchanged MPXI in human viral infections was thought to be because lymphocytes, and not neutrophils, are the main effector immune cells in viral infections.^63^ However, this finding is unlikely to translate to CPE because neutrophils play an important role in anti-viral defenses, and the severe gastrointestinal inflammation leads to the recruitment and activation of large numbers of neutrophils.^18,31^

Third, a previous study found that humans with non-septic bacterial infections had increased MPXI values, whereas septic patients had decreased MPXI values.^63^ Therefore, it was postulated that more severe bacterial infections would result in increased neutrophil activation and degranulation, leading to lower MPXI values.^63^ Conversely, endotoxin tolerance or immunoparalysis, a phenomenon of reduced neutrophil function after exposure to endotoxins, has been documented in septic humans and dogs.^5,47,61^ One characteristic of reduced neutrophil function is lower phagolysosomal oxidative burst activity,^47,61^ a process in which MPO is a vital component.^26^ This mechanism is supported by the reduced oxidative burst activity evident in neutropenic dogs with CPE.^13^ Consequently, impaired neutrophil function associated with endotoxin tolerance may lead to retention of intracellular MPO, thereby contributing to increased MPXI in dogs with CPE.

Fourth, apart from immunoparalysis, direct viral-induced suppression of neutrophil function, as demonstrated in human influenza virus and Dengue virus infections, may also increase MPXI.^23,36^

Last, a high MPXI may be encountered in severe inflammatory conditions when immature neutrophils are released into circulation before completion of all cellular divisions.^55,65^

As discussed, viral- and endotoxin-induced inhibition of oxidative burst activity may lead to increased MPXI values because of increased neutrophil MPO content. This mechanism is supported by the strong positive correlations observed between MPXI and both CRP and cortisol concentrations, as well as the concurrent leukopenia identified in affected dogs, which together suggest the presence of systemic inflammation, stress response, and reduced neutrophil function. A 2024 report further supports this interpretation. Compared with survivors, non-surviving dogs with CPE had significantly higher MPXI values on days 2, 4, and 5 of hospitalization, with markedly higher MPXI values on day 5.^58^ The progressive increase in MPXI observed in non-survivors aligns with the concept of immunoparalysis secondary to endotoxin exposure. Given the pathophysiology of CPE (and the associated risks of bacterial translocation, sepsis, and SIRS), immunoparalysis is the most likely mechanism contributing to the increases in MPXI that we observed.^40,51^

Dogs with CPE had significantly lower WBC counts compared with healthy controls, and, unexpectedly, MPXI had a strong inverse correlation with the WBC count, particularly at T1. Leukopenia, with neutropenia and lymphopenia, is a hallmark of CPE and increases the risk of bacterial translocation, sepsis, and death.^18,51^ Higher WBC counts following treatment have been shown to predict survival.^3,15,18^ In our study, the negative correlation suggests that increasing MPXI may be associated with more severe leukopenia and, by extension, a worse prognosis.

At admission, neutrophil counts did not differ significantly between CPE-infected and control dogs; however, counts at T1 and T2 were significantly lower in affected dogs. Surprisingly, the MPXI was inversely correlated with the neutrophil count, with the strongest correlation at T1. Neutropenia in CPE results from recruitment of neutrophils at the site of infection; destruction of hematopoietic progenitor cells in the bone marrow, thymus, spleen, and lymph nodes; increased neutrophil demand in inflamed intestines; and endotoxemia prompting neutrophil margination.^12,31,51^ Clinical signs have been reported to precede the onset of neutropenia by one day, with the neutrophil nadir occurring when clinical disease was at its worst.^12^ In our study, prospective cases had a median duration of illness of 1 d (range: 1–3 d) before presentation, indicating that the patients likely were early in the course of the disease, before the onset of neutropenia. Prognostically, lower neutrophil counts have been associated with mortality; increasing counts over time are predictive of survival.^3,15,18^ Therefore, the negative correlation found between MPXI and the neutrophil count may indicate that increased MPXI mirrors worsening neutropenia and, therefore, poorer outcomes.

Lymphocyte counts were significantly lower in CPE dogs. The MPXI was correlated negatively with the lymphocyte count, with the strongest correlation seen at T1. Lymphopenia is attributed to cortisol-mediated sequestration within lymph nodes and to viral destruction of lymphocytes and lymphoid progenitors.^50,51^ As with leukopenia and neutropenia, lymphopenia is associated with a worse prognosis in dogs with CPE,^3,18^ and the negative correlation with MPXI further supports this index as a potential marker of disease severity.

CRP concentrations were significantly increased in affected dogs and positively correlated with MPXI, particularly at T1. We hypothesized that a negative correlation would exist between MPXI and CRP. However, given our finding of an increasing MPXI in CPE dogs, and the possible pathophysiology behind the increase, the positive correlation we observed is not surprising. Another possible reason for our finding is that MPXI may be similarly increased in response to inflammation depending on the stage of neutrophil maturation and degranulation.

Total thyroxine concentrations were significantly lower in CPE dogs and negatively correlated with MPXI. In humans, suppression of the hypothalamic-pituitary-thyroid axis by pro-inflammatory cytokines is considered a major cause of non-thyroidal illness syndrome (NTIS).^14,46^ NTIS is common in CPE, and persistently low thyroxine levels are associated with non-survival.^42,54^ Hence, the negative association of MPXI with thyroxine further supports its possible prognostic role in dogs with CPE.

Cortisol concentrations were significantly increased in affected dogs and strongly, positively correlated with MPXI at both T1 and T2. During physiologic stress (e.g., exercise, trauma, infection), the cortisol concentration increases, with the magnitude of increase proportional to the severity of the stressor.^32,39^ Studies have shown that persistently increased cortisol concentrations are associated with increased mortality and prolonged hospitalization in dogs with CPE.^15,54^ Therefore, the positive association of MPXI with cortisol further underscores its role as a potential marker of disease severity and outcome in dogs with CPE.

Our study has several limitations. Inclusion was initially limited to hospitalized dogs, potentially introducing a population bias. Unfortunately, prospective recruitment was poor over 18 mo, which is likely a reflection of successful vaccination strategies as well as the lack of affordability of in-hospital treatment. Consequently, the prospective cohort may have been underpowered, hampering detection of a significant difference in MPXI between the control population and the diseased dogs. Therefore, we included retrospective data for analysis, which did not contribute to the data for evaluation at T1 and T2. Furthermore, the few mortalities recorded precluded evaluation of MPXI differences between survivors and non-survivors, preventing evaluation of the prognostic ability of MPXI. Two different hematology analyzers were used across sites, which may serve as a source of measurement bias. However, these analyzers were comparable when assessed against hematology ASVCP TEa guidelines. Delays in CBC analysis of up to 12 h and serum sample transport and/or storage at 4°C for up to 5 d for samples collected at PVC may have affected MPXI and biomarker stability, although literature suggests minimal degradation within that storage timeframe.^2,11,44,52^ Finally, long-term frozen storage of retrospective samples could have impacted cortisol measurements, because long-term stability has not been verified.
